# Biodegradable Nanocomposite Films Based on Sodium Alginate and Cellulose Nanofibrils

**DOI:** 10.3390/ma9010050

**Published:** 2016-01-14

**Authors:** B. Deepa, Eldho Abraham, Laly A. Pothan, Nereida Cordeiro, Marisa Faria, Sabu Thomas

**Affiliations:** 1 Department of Chemistry, Bishop Moore College, Mavelikara-690101, Kerala, India; deepabkrishnan@gmail.com; 2 Department of Chemistry, Church Missionary Society(C.M.S) College, Kottayam-686001, Kerala, India; 3 Robert H. Smith Faculty of Agriculture, Food and Environment, Hebrew University, Jerusalem, POB 12, Rehovot 76100, Israel; eldhoabraham@gmail.com; 4 Faculty of Exact Science and Engineering, University of Madeira, 9000-390 Funchal, Portugal; ncordeiro@uma.pt (N.C.); marisa.faria@uma.pt (M.F.); 5 School of Chemical Sciences, Mahatma Gandhi University, Kottayam-686560, Kerala, India; sabuchathukulam@yahoo.co.uk

**Keywords:** cellulose nanofibrils, sodium alginate, nanocomposite films, solvent casting, mechanical properties

## Abstract

Biodegradable nanocomposite films were prepared by incorporation of cellulose nanofibrils (CNF) into alginate biopolymer using the solution casting method. The effects of CNF content (2.5, 5, 7.5, 10 and 15 wt %) on mechanical, biodegradability and swelling behavior of the nanocomposite films were determined. The results showed that the tensile modulus value of the nanocomposite films increased from 308 to 1403 MPa with increasing CNF content from 0% to 10%; however, it decreased with further increase of the filler content. Incorporation of CNF also significantly reduced the swelling percentage and water solubility of alginate-based films, with the lower values found for 10 wt % in CNF. Biodegradation studies of the films in soil confirmed that the biodegradation time of alginate/CNF films greatly depends on the CNF content. The results evidence that the stronger intermolecular interaction and molecular compatibility between alginate and CNF components was at 10 wt % in CNF alginate films.

## 1. Introduction

Recently, bio-nanocomposite films developed from biopolymers have gained considerable attention due to their renewability, biodegradability, biocompatibility, low toxicity and their potential use in the packaging industry. In this context, studies based on two such biopolymers, namely cellulose and sodium alginate, have received renewed interest. Cellulose is the most abundant renewable biopolymer on earth and it is a polydispersed linear polymer of poly-β(1,4)-d-glucose units [1]. The two novel forms of cellulose, namely cellulose nanofibrils (CNF) and cellulose nanocrystals (CNC), appear to be attractive building blocks to produce high performance bionanomaterials. They exhibit outstanding properties such as low density, low thermal expansion, high aspect ratio and important mechanical properties, which make them relevant in various applications [2,3,4,5,6].

Alginate is another natural biopolymer, composed mainly of (1,4)-linked β-d-mannuronic acid units and α-l-guluronic acid units [7,8,9]. The main source of alginate is the cell wall of brown algae, and for commercial purposes alginate is extracted from seaweed. Alginates and alginate-based biocomposites have been used in food packaging, tissue engineering, biomedicine and pharmaceutical fields due to their non-toxicity, biodegradability, biocompatibility and unique gel-forming characteristics [8]. It has also been used for the preparation of biodegradable films. However, poor mechanical and weak water resistance properties limit its application, particularly in the presence of water and humidity [10,11]. The present study attempted to overcome these problems by developing nanocomposite films with the incorporation of cellulose nanofibrils into the alginate matrix. Cellulose nanofibrils used for this study were extracted from sisal fibers by steam explosion coupled with the acid hydrolysis process. The objective of this study was to investigate the effect of cellulose nanofibrils (CNF) at various loadings on the mechanical, biodegradability and swelling behavior of the resulting nanocomposite films.

## 2. Results and Discussion

### 2.1. Mechanical Properties

**Figure 1:**
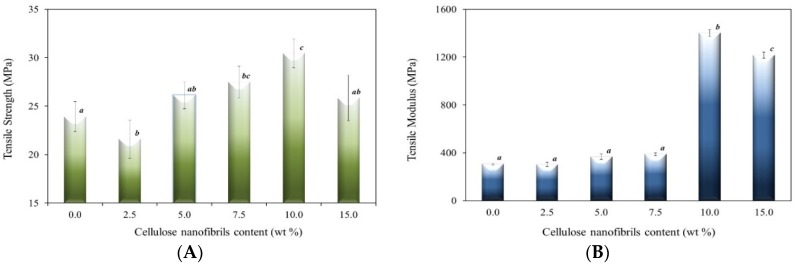
(**A**) Tensile strength and (**B**) Tensile modulus of cellulose nanofibril (CNF)-reinforced alginate films as the function of the CNF content (lower case letters (a, b, c) show Duncan grouping; distinct letters represent means significantly different (*p* < 0.05)).

The effect of CNF loading on the tensile properties of alginate films is presented in Figure 1. In general, we observed an improvement of the mechanical properties, attributed to the good interfacial interaction between CNF and the alginate matrix due to the similar polysaccharide structures [10] and to the surface acid-base character (discussed below) of cellulose and alginate matrix.

**Figure 2:**
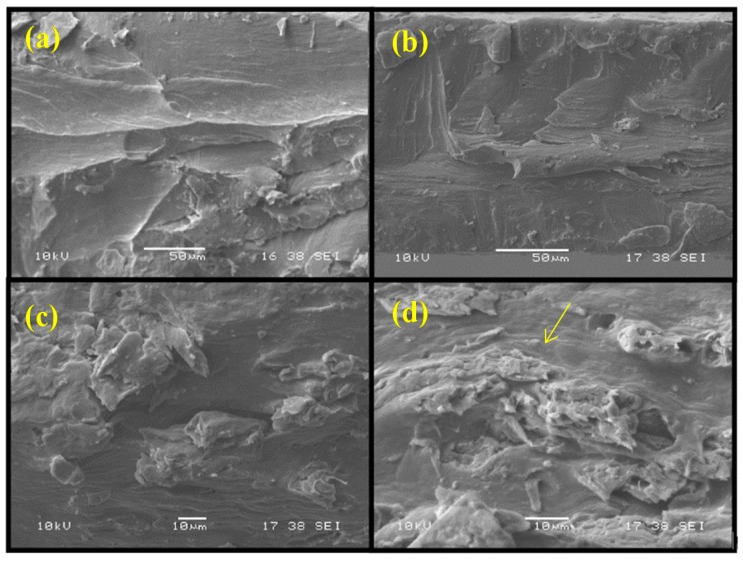
Scanning electron microscopy images of cross-section of the (**a**) alginate; (**b**) alginate with 5 wt % CNF; (**c**) alginate with 10 wt % CNF; and (**d**) alginate with 15 wt % CNF films (arrow indicates the CNF agglomeration in alginate matrix).

It has been noted that the incorporation of 2.5 wt % of CNF negatively influences the mechanical properties of the alginate films. This can be attributed to the (i) destruction of the alginate chain organization, which weakens the composite and was not counterbalanced by the few links stabilized between the CNF and the alginate chains, or to the (ii) failure strain difference between the CNF and the matrix [12]. In other words, the reinforcement of CNF does not have an effect when the failure strain of the matrix polymer is much greater than that of the nanocellulose. So, the nanocomposite shows a failure before the stress is transferred to the CNF from the matrix [12]. At lower CNF content (≤10 wt %), CNF could disperse well in the alginate matrix, which increases the mechanical properties (tensile strength (TS) and tensile modulus(TM)) of the nanocomposite. However, at higher content (>10 wt %), the aggregation of CNF (observed by the Scanning electron microscopy (SEM) images, Figure 2) causes a poor dispersion of CNF in the alginate matrix [9]. As a result, both the tensile strength and tensile modulus of the films decrease for CNF additions higher than 10 wt %.

The tensile strength decreases at 2.5 wt %, and then increases with an increase in the CNF content up to 10 wt %, followed by a decrease at higher CNF content (Figure 1a). Similar behavior was observed in the tensile modulus of CNF-reinforced alginate films (Figure 1b). The tensile modulus (TM) value of the pure alginate film was found to be around 308 MPa. The incorporation of CNF into the alginate matrix caused a significant (*p* < 0.05) increase of TM to 1403 MPa, which is around 356% higher than that of the pure alginate film. The highest TM was observed for 10% CNF loading. The increased TM values of the alginate films obtained after the incorporation of CNF may be attributed to the increased stiffness of the films by the addition of nanocellulose. It also reveals the high reinforcing efficiency of CNF material in the alginate matrix.

For the tensile modulus, it was not observed the expected linear increase with the increase of CNF content; instead we observed a decrease at 2.5 wt % (above-mentioned) and a very high increase at 10 wt % of CNF incorporation. Given the surface nature of the CNF and alginate chain (later debated in the Inverse Gas Chromatography (IGC) analysis, this may be due to an optimization of the interactions between the chains of the CNF and alginate matrix, with the formation of a network-like structure increasing the TM significantlyto the 10 wt % of CNF incorporation.

### 2.2. Fourier Transform Infrared Spectroscopy (FTIR) Analysis

**Figure 3:**
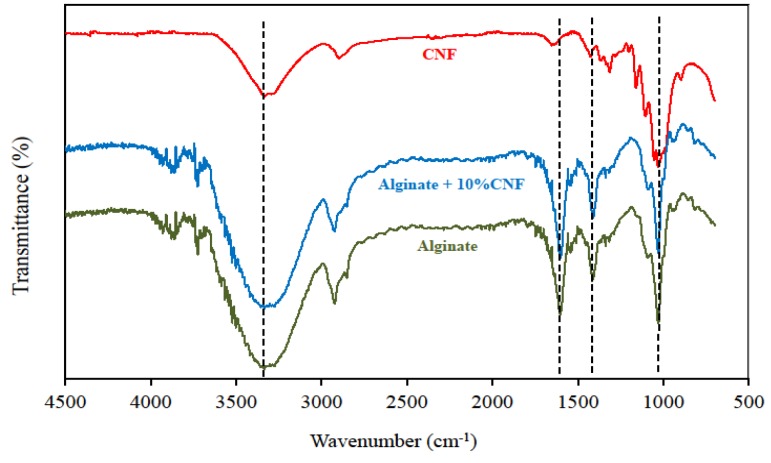
Fourier Transform Infrared (FTIR) spectra of cellulose nanofibrils (CNF), alginate film, alginate with 10 wt % of CNF film.

Fourier Transform Infrared Spectroscopy (FTIR) analysis of films attempted to characterize the incorporation of CNF into the alginate-based film matrix by distinguishing the infrared (IR) bands and vibration shifts related to the CNF-alginate interactions (Figure 3). The characteristic peak of alginate is located at 1602 cm^−1^, corresponding to the carbonyl bond (–C=O). The peaks at 1424 and 1024 cm^−1^ in both alginate and cellulose were attributed to carboxyl stretching bands (–COO and –C–O) [13]. In addition, peaks at 3334 and 2930 cm^−1^ were ascribed to the–O–H and –C–H of aliphatic chain stretching vibrations, respectively [13,14]. Some differences can be observed after CNF addition into the alginate matrix. A slight increase of the intensity and width of the overall –O–H band was observed as the CNF was incorporated into the alginate matrix. This can indicate an increase of hydrogen bonding between the alginate and CNF [15]. Overall, FTIR spectra of cellulose-loaded alginate films provided qualitative insights into the effect of cellulose concentration on the position, width and intensity of IR vibrations related to alginate-cellulose interactions, although many bands from the alginate spectra masked typical cellulose vibrations, especially in the 1800–1270 cm^−1^ region (implying bands related to the degree of order of cellulosic materials) [9]. All these changes support the evidence of intermolecular interactions and molecular compatibility between alginate and cellulose components.

### 2.3. Inverse Gas Chromatography (IGC) Analysis

In order to understand the nature of alginate/CNF interactions and their effect on surface properties, the films were analyzed by Inverse Gas Chromatography (IGC). Through non-polar and polar probe molecules, the dispersive component of surface energy ($\gamma_{s}^{d}$) and the specific component of surface energy ($∆G_{S}^{sp}$) were determined, respectively. Table 1 includes the obtained IGC results. The $\gamma_{s}^{d}$ found shows that the interaction between alginate/CNF makes a slightly less dispersive film surface.

**Table 1:** Dispersive component of the surface energy ($\gamma_{s}^{d}$), acid (*K*_a_) and base (*K*_b_) constants and acid/base ratio (*K*_a_/*K*_b_) of the cellulose nanofibrils (CNF) and alginate films at 25 °C.

Sample	$\gamma_{s}^{d}$ (mJ/m^2^) *	*K*_a_	*K*_b_	*K*_a_/*K*_b_
CNF	39.79	0.08	0.20	0.4
Alginate	38.66	0.09	0.05	1.8
Alginate with 10 wt % CNF	36.87	0.09	0.04	2.3

* Correlation coefficient between 0.998 and 0.999.

**Figure 4:**
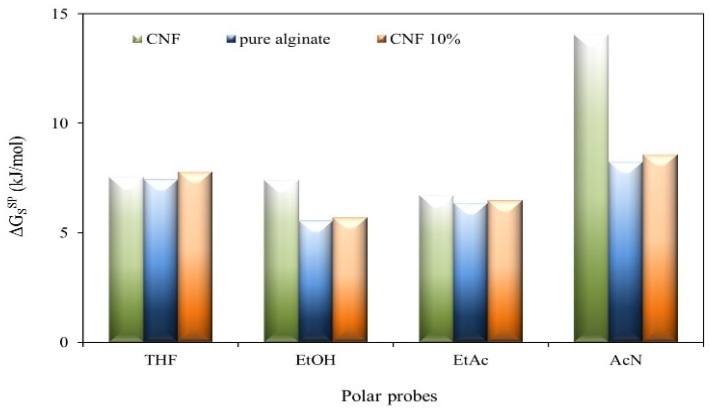
Specific surface free energy ($∆G_{S}^{sp}$) obtained for cellulose nanofibrils (CNF) and alginate films at 25 °C.

The $∆G_{S}^{sp}$ was determined by IGC (Figure 4) using four polar probes: tetrahydrofuran (THF), ethanol (EtOH), ethyl acetate (EtAc) and acetonitrile (AcN). A more significant increase in the $∆G_{S}^{sp}$ was observed for the THF probe (base probe) due to CNF incorporation in the alginate matrix. This can indicate a more acidic surface in the alginate/CNF composites.

**Figure 5:**
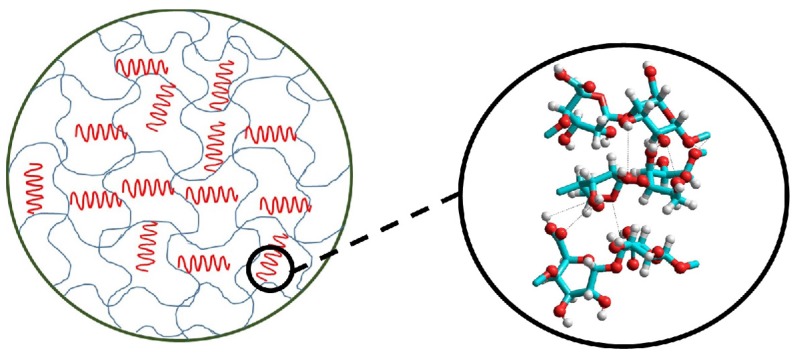
Schematic representation of the interaction between cellulose nanofibrils (CNF) and alginate matrix.

The obtained values of $∆G_{S}^{sp}$ were converted into acid-base constants (*K*_a_ and *K*_b_) and the results (Table 1) lead to the conclusion that the interaction between CNF and alginate takes place preferentially via the hydrogen bond between the oxygen carboxylic (basic group) and the OH from the CNF cellulose. Furthermore, this interaction probably causes a reorganization in the alginate chain and exposes more acid groups (OH) to the surface, as schematized in Figure 5.

### 2.4. Water Solubility

Solubility in water is an important property of films for food packaging applications. Some potential uses may require water insolubility to enhance product integrity and water resistance. However, in other cases, water solubility (WS) of the film before product consumption might be useful as in the encapsulation of food or additives.

**Figure 6:**
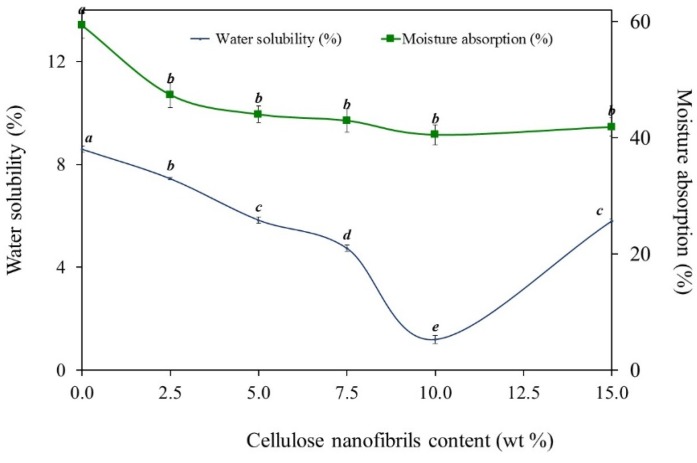
Effect of CNF content on the moisture absorption and water solubility of alginate/CNF films (lower case letters (a, b, c, d, e) show Duncan grouping; distinct letters represent means significantly different (*p* < 0.05)).

WS of alginate-based films as a function of CNF addition is given in Figure 6. The addition of CNF significantly reduces (*p* < 0.05) the film water solubility from 8.6% to 1.7% (10 wt % CNF). It is indicative of a strong hydrogen bond formation between the CNF and the film matrix. The hydroxyl groups of CNF can form strong interactions through hydrogen bonds with the hydroxyl and carboxyl groups on alginate and improve the cohesiveness of the biopolymer matrix while decreasing the water sensitivity. These interactions provide stability and resistance to alginate/CNF films. Inother words, water molecules cannot break these strong bonds sufficiently, thus the WS is decreased. Rhim [16] reported that the reduction observed in solubility of films reinforced with different nanofillers is mainly related to the strong hydrogen bond formation between hydroxyl groups of the biopolymer and the nanoparticles. It has also been reported that the high crystallinity of CNF (in this study, 91.3%) and their strong hydrogen-bonded networks within the polymer matrix were responsible for the water resistance improvement [17].

### 2.5. Moisture Absorption

Water sensitivity of the alginate/CNF films is also evaluated by monitoring the moisture absorption at different time intervals (Figure 6). The experimental results obtained indicate that the incorporation of CNF reduces moisture absorption, demonstrating that water has less affinity with the film. These results corroborate the behavior observed in water solubility tests. CNF presents a stronger interaction with alginate than with water. The number of active sites for water binding decreases due to the formation of strong bonds between the alginate matrix and CNF, reducing the moisture content of alginate/CNF films.

### 2.6. Swelling Behaviour

**Figure 7:**
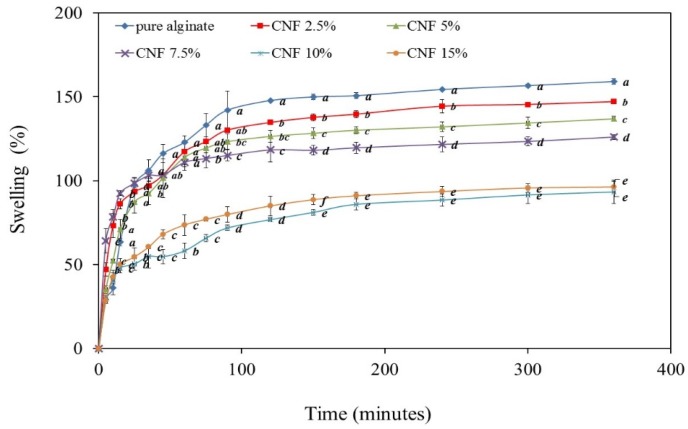
Effect of CNF content on the swelling ratio of alginate/CNFfilms (lower case letters (a, b, c, d, e) show Duncan grouping; distinct letters represent means significantly different (*p* < 0.05)).

The swelling behavior of CNF-reinforced alginate-based films is presented in Figure 7. Incorporation of CNF significantly reduced the swelling percentage (SW) of alginate-based films, with the most significant decrease at 10 wt % CNF incorporation, decreasing about 55% after 6 h of immersion in water when compared with pure alginate films. After 1 h, the SW value of the pure alginate films was found to be 124%, whereas due to the incorporation of 10% and 15% CNF, the SW values of the nanocomposite films were found to be 65% and 77%, respectively.

### 2.7. Biodegradability

**Figure 8:**
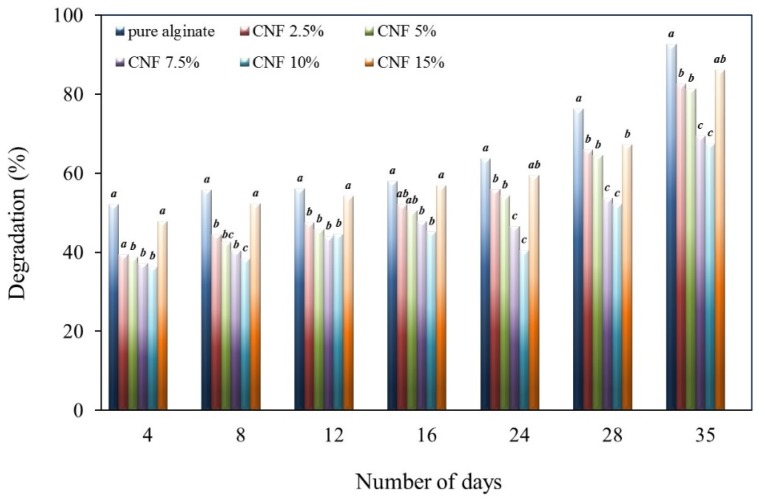
Effect of CNF content on the rate of degradation of alginate/CNF films (lower case letters (a, b, c) show Duncan grouping; distinct letters represent means significantly different (*p* < 0.05)).

Biodegradability is another important property for films in food packaging. Biodegradability of pure alginate and CNF-reinforced alginate films were studied. Figure 8 shows the effect of CNF content on the rate of degradation of alginate/CNF films in compost. It is observed that the biodegradability of pure alginate films increased up to 90% as the burial time increased in the compost for five weeks. However, in the case of CNF-reinforced alginate films, the rate of degradation changes with varying amounts of CNF added to the alginate matrix.

When CNF is added to the alginate matrix, there arises a strong interaction between the matrix and the filler due to the homogeneous dispersion of CNF particles in the alginate matrix. Thus, the degradation of films becomes difficult due to difficulty in breaking the strong bonds between the alginate matrix and nanocellulose. Therefore, it can be concluded from the results that the CNF incorporation in alginate matrix decreases the degradation percentage of films up to 10 wt % of CNF. However, at higher concentrations of CNF (15 wt % of CNF), a reverse effect is observed due to agglomeration of CNF particles in the alginate matrix and the decrease in the alginate/CNF interaction.

## 3. Experimental Section

### 3.1. Materials

Sodium alginate used in this study was obtained from LobaChemie Pvt. Ltd. (Mumbai, India). Glycerol, sodium hydroxide, acetic acid, sodium chlorite and oxalic acid were purchased from Merck Pvt. Ltd. (Mumbai, India). Cellulose nanofibrils (CNF) used were isolated from sisal fibers by steam explosion process (percentage yield of 39%) as described and characterized in Deepa *et al*. (2015) [18]. Atomic force microscopy images (AFM) revealed a high value (5.3 nm) of the surface roughness, which predict a good interfacial adhesion between the CNF and the matrix in the composites preparation. The diameters of the nanocellulose structures were found to be between 20–80 nm with an average width of 13 ± 4 nm. Transmission electron microscopy (TEM) images showed that CNF were long, flexible, entangled, with several micrometers in length. A very high aspect ratio (length/diameter) was found as important parameter to obtain composites with good mechanical properties. By *X*-ray Diffraction (XRD) analysis, the crystallinity index and the crystal size of CNF were calculated to be 81.6% and 3.7 nm, respectively.

### 3.2. Films Preparation

A solution of sodium alginate was prepared by dissolving the sodium alginate (3% w/w) in distilled water at 60–70 °C under stirring for 30 min. After complete dissolution, 0.5 g glycerol/1 g alginate was added as a plasticizer to enhance film flexibility, decrease brittleness and facilitate their detachment from the petri dishes. The film-forming solution was cast onto petri dishes which were 14cm in diameter and oven dried at 40 °C for approximately 24 h. The dried films were then removed from the petri dishes and stored in polyethylene bags prior to characterization.

In order to prepare the nanocomposite films, different amounts of the cellulose nanofibrils (CNF), 2.5%, 5%, 7.5%, 10% and 15% (w/w) on solid sodium alginate, were added to 30 mL of deionized water and dispersed/homogenized using a homogenizer at 10,000 rpm for 30 min at room temperature. Afterwards, the CNF solution was added slowly to the alginate solutions (made as described above). The resulting mixture was stirred for 1 h. Finally, the films were prepared and stored, as described previously.

### 3.3. Mechanical Properties

Mechanical properties of the film samples including tensile strength (TS) and tensile modulus (TM) were measured according to standard method ASTM D882-02 [19] using an Instron Universal Testing Machine equipped with a 100 N-load cell. Prior to the test, the film samples were cut into rectangular strips (15 mm × 70 mm × 0.2 mm). The initial grip separation was set at 50 mm and crosshead speed at 50 mm/min. An average value was taken from the measurements of five films under the same conditions for each specimen.

### 3.4. Scanning Electron Microscopy (SEM)

Scanning electron microscopy (SEM) images of film samples were obtained using a Scanning Electron Microscope model JEOL JSM-6390LV (JEOL Ltd., Tokyo, Japan) with an accelerating voltage of 10–20 kV. Prior to SEM examination, the samples were oven dried at 60 °C for 8 h and then a fine layer of gold was deposited on samples by means of a plasma sputtering apparatus.

### 3.5. Fourier Transform Infrared Spectroscopy (FTIR)

Infrared spectra (4000–640 cm^−1^) of the film samples were recorded on a Fourier Transform Infrared Spectrometer Perkin-Elmer (PC1600, Perkin-Elmer, Waltham, MA, USA) equipped with an Attenuated Total Reflectance (ATR) device with a spectral resolution of 4 cm^−1^ and after 32 scans.

### 3.6. Inverse Gas Chromatography (IGC)

Surface properties were analyzed using a commercial inverse gas chromatography (Surface Measurements Systems, London, UK). The measurements and theory applied were described exhaustively by Cordeiro [20]. In this study, all the measurements were carried out at 0% relative humidity (RH), a helium flow rate of 10 mL/min and in duplicate producing an experimental variation below 4%. Methane was used as reference molecule. Heptane, octane, nonane and decane were the four *n*-alkanes used for determination of the dispersive component of the surface free energy at 25 °C. Acetonitrile (ACN), ethyl acetate (EtOAc), ethanol (EtOH) and tetrahydrofuran (THF) were used for determination of the Gibbs specific free energy ($∆G_{S}^{SP}$) and acid-base surface character (*K*_A_ and *K*_B_) at 25 °C.

### 3.7. Water Solubility

The solubility of the films in water was determined as percentage of film dry matter solubilized in water during a period of 24 h was described by Gontard [21]. The oven-dried film samples were cut (20 mm × 20 mm × 0.2 mm), weighed, immersed into 20 mL distilled water and slowly agitated. The amount of dry matter in the initial (*W_i_*) and final (*W_f_*) samples was determined by drying the samples at 105 °C. The film water solubility (*WS*) was calculated using the Equation (1):

(1) $$WS\left(\%\right)=\frac{W_{i}-W_{f}}{W_{i}}\times 100$$

### 3.8. Moisture Absorption

The moisture absorption of the films was determined after drying in an oven at 105 °C for 24 h, according to ASTM D5229 standard methodology [19]. Briefly, the dry films were conditioned at 25 °C in desiccators containing oversaturated solutions of sodium sulfate in order to ensure a relative humidity (RH) of 95%. The samples were removed at specific time and the rate of moisture absorption (*MA*) was calculated by the Equation (2):

(2) $$MA\left(\%\right)=\frac{W_{t}-W_{i}}{W_{i}}\times 100$$

where *W_t_* and *W_i_* represents the film weight at time *t* and before exposure to 95% RH, respectively.

### 3.9. Swelling Behavior

The swelling behavior of the films was described by the swelling ratio (*SW*) determined according to standard method ASTM D2765-95C [19]. The swelling ratio (*SW*) of the films was calculated using the Equation (3):

(3) $$SW\left(\%\right)=\frac{W_{s}-W_{d}}{W_{d}}\times 100$$

where *W_s_* is the samples weight after swelling, and *W_d_* is the weight of dry sample.

### 3.10. Biodegradation Analysis

Soil burial tests for biodegradation of the film samples were carried out by a method described by Martucci [22]. The samples were cut into rectangular pieces (20 mm × 20 mm), dried in an oven at 105 °C for 24 h and weighed (*W_i_*). The films were then buried under compost in plastic boxes (120 mm × 60 mm × 1.5 mm) at a depth of 8 cm from the soil surface in order to ensure aerobic degradation conditions. The assay was performed at 32 °C and 35% relative humidity (RH) by adding water periodically. Fluctuations in soil moisture were followed gravimetrically using the standard method of oven drying (ASTM D2216) [19]. Samples were taken from the soil at different times and cleaned by wiping gently with a tissue paper. Subsequently, they were dried in an oven at 105 °C for 12 h and weighed (*W_t_*) to determine the % of Weight Loss (*WL*) using the Equation (4):

(4) $$WL\left(\%\right)=\frac{W_{i}-W_{t}}{W_{i}}\times 100$$

### 3.11. Statistical Analysis

All experiments were carried at least in duplicate. Analysis of variance (ANOVA) in SPSS statistics software (Version 23; SPSS Inc., Chicago, IL, USA) followed by Duncan’s test were used for statistical analysis of data. *p*-values < 0.05 were considered statistically significant.

## 4. Conclusions

Cellulose nanofibrils (CNF) isolated from sisal fiber by steam explosion coupled with the acid hydrolysis process was used to prepare nanocomposite films from alginate biopolymer. The similar chemical structures of the alginate matrix and the CNF led to strong adhesion between them through hydrogen bonding. In general, the results indicate that the biofilm alginate with 10% CNF presents the best properties. The incorporation of CNF into the alginate matrix improved its water resistance and mechanical properties. Swelling and water solubility measurements indicated that CNF-reinforced alginate films have less affinity towards water molecules. The biodegradability of the films investigated by the soil burial method demonstrated that the biodegradation time of alginate/CNF films was longer than that of pure alginate films. These nanocomposite films present excellent potential as a new biomaterial for application in food packaging and conservation.

## References

[1] Klemm D., Kramer F., Moritz S., Lindstrom T., Ankerfors M., Gray D., Dorris A. (2011). Nanocelluloses: A new family of naturebased materials. Angew. Chem. Int. Ed..

[2] Abdul Khalil H.P.S., Davoudpour Y., Islam M.N., Mustapha A., Sudesh K., Dungani R., Jawaid M. (2014). Production and modification of nanofibrillated cellulose using various mechanical processes: A rewiew. Carbohydr. Polym..

[3] Sacui I.A., Nieuwendaal R.C., Burnett D.J., Stranick S.J., Jorfi M., Weder C., Foster E.J., Olsson R.T., Gilman J.W. (2014). Comparison of the properties of cellulose nanocrystals and cellulose nanofibrils isolated from bacteria, tunicate, and wood processed using acid, enzymatic, mechanical, and oxidative methods. ACS Appl. Mater. Interfaces.

[4] Habibi Y., Lucia L.A., Rojas O.J. (2010). Cellulose nanocrystals: Chemistry, self-assembly, and applications. Chem. Rev..

[5] Eichhorn S.J., Dufresne A., Aranguren M., Marcovich N.E., Capadona J.R., Rowan S.J., Weder C., Thielemans W., Roman M., Renneckar S. (2010). Review: Current international research into cellulose nanofibresand nanocomposites. J. Mater. Sci..

[6] Azzam F., Moreau C., Cousin F., Menelle A., Bizot H., Cathala B. (2014). Cellulose nanofibril-based multilayered thin films: Effect of ionic strength on porosity, swelling, and optical properties. Langmuir.

[7] Norajit K., Kim K.M., Ryu G.H. (2010). Comparative studies on the characterization and antioxidant properties of biodegradable alginate films containing ginseng extract. J. Food Eng..

[8] Abdollahi M., Alboofetileh M., Rezaei M., Behrooz R. (2013). Comparing physico-mechanical and thermal properties of alginate nanocomposite films reinforced with organic and/or inorganic nanofillers. Food Hydrocoll..

[9] Huq T., Salmieri S., Khan A., Khan R.A., Le Tien C., Riedl B., Fraschini C., Bouchard J., Uribe-Calderon J., Kamal M.R. (2012). Nanocrystalline cellulose (NCC) reinforced alginate based biodegradable nanocomposite film. Carbohydr. Polym..

[10] Abdollahi M., Alboofetileh M., Behrooz R., Rezaei M., Miraki R. (2013). Reducing water sensitivity of alginate bio-nanocomposite film using cellulose nanoparticles. Int. J. Biol. Macromol..

[11] Rhim J.W. (2004). Physical and mechanical properties of water resistant sodium alginate films. LWT Food Sci. Technol..

[12] Cho M.J., Park B.D. (2011). Tensile and thermal properties of nanocellulose-reinforced poly (vinyl alcohol) nanocomposites. J. Ind. Eng. Chem..

[13] Lin N., Bruzzese C., Dufresne A. (2012). Tempo-oxidized nanocellulose participating as crosslinking aid for alginate-based sponges. ACS Appl. Mater. Inter..

[14] Han J., Guenier A.S., Salmieri S., Lacroix M. (2008). Alginate and chitosan functionalization for micronutrient encapsulation. J. Agric. Food Chem..

[15] Khan R.A., Salmieri S., Dussault D., Uribe-Calderon J., Kamal M.R., Safrany A., Lacroix M. (2010). Production and properties of nanocellulose-reinforced methylcellulose-based biodegradable films. J. Agric. Food Chem..

[16] Rhim J.W., Ng P.K.W. (2007). Natural biopolymer-based nanocomposite films for packaging applications. Crit. Rev. Food Sci..

[17] De Paula E.L., Mano V., Pereira F.V. (2011). Influence of cellulose nanowhiskers on the hydrolytic degradation behavior of poly (d, l-lactide). Polym. Degrad. Stabil..

[18] Deepa B., Abraham E., Cordeiro N., Mozetic M., Mathew A.P., Oksman K., Faria M., Thomas S., Pothan L.A. (2015). Utilization of various lignocellulosic biomass for the production of nanocellulose: A comparative study. Cellulose.

[19] (1997). ASHRAE Handbook, Fundamentals.

[20] Cordeiro N., Gouveia C., Moraes A.G.O., Amico S.C. (2011). Natural fibres characterization by inverse gas chromatography. Carbohydr. Polym..

[21] Gontard N., Guilbert S., Cuq J.L. (1992). Edible wheat gluten films: Influence of the main process variables on film properties using response surface methodology. J. Food Sci..

[22] Martucci J.F., Ruseckaite R.A. (2009). Biodegradation of three-layer laminate films based on gelatin under indoor soil conditions. Polym. Degrad. Stabil..

